# Towards Streamlined Transparent Data Linkage

**DOI:** 10.23889/ijpds.v9i5.2575

**Published:** 2024-09-18

**Authors:** Michael Edwards, Alexandra Lee, Simon Thompson

**Affiliations:** 1 Swansea University

In recent years, great strides have been made towards the deployment of federated systems for data research, including exploring federated trusted research environments (TREs). These federated TREs allow data analysts to securely use data within multiple institutions with minimal overheads while preserving information governance. However, due to the sensitive nature of data utilised in data linkage pipelines, especially within privacy-preserving applications, the real-world deployment of federated data linkage is in its early stages.

There are several challenges to overcome with privacy-preserving federated linkage, including securely and efficiently sharing information that enables the identification of record similarities. Another key consideration is the resilience of the federated linkage ecosystem to information being added or removed as data providers move in and out of the initiative, as changes in the record set can cause intermediary links to be destroyed or created. Within non-federated linkage, a spine dataset such as a national census can provide an anchor on which incoming datasets can be linked. In federated linkage this may not explicitly be the case, especially if providers are globally dispersed or such a broad spine does not exist for the use case, and so there may be critical datasets within the overall system.

We present a discussion over the real-world challenges to federated linkage on population-scale applications, including approaches which allow privacy-preserving linkage across multiple TREs and handling information governance requirements. We also explore the impact of the collaborative nature of federated linkage, looking at how quality and record links are affected in practice.

